# Access to Healthcare and Health Literacy in Croatia: Empirical Investigation

**DOI:** 10.3390/healthcare11131955

**Published:** 2023-07-06

**Authors:** Ana Bobinac

**Affiliations:** Center for Health Economics and Pharmacoeconomics (CHEP), Faculty of Economics and Business, University of Rijeka, 51 000 Rijeka, Croatia; ana.bobinac@efri.hr

**Keywords:** health literacy, access to care, unmet medical need

## Abstract

Health literacy is related to different health-related outcomes. However, the nature of the relationship between health literacy and health outcomes is not well understood. One pathway may lead from health literacy to health outcomes by means of access to healthcare. The goal of the current study is to explore the association between health literacy and the particular measure of access to healthcare—unmet medical need—for the first time in Croatia and, to the best of our knowledge, for the first time in the EU context. We use data obtained from face-to-face interviews in a large nationally representative sample of the Croatian population (*n* = 1000) to estimate the level of health literacy and self-reported access to care and investigate the association between health literacy and self-perceived barriers to access. Our study showed that limited and problematic health literacy is prevalent and associated with higher rates of unmet medical need. Unmet need is largely caused by long waiting lists. It is therefore essential to design health services fitting the needs of those who have limited and/or problematic health literacy as well as enhance health education with the potential of improving the access to care and health outcomes as well as design policies that reduce waiting times.

## 1. Introduction

Health literacy is a multidimensional concept conventionally defined as the degree to which individuals have the capacity to attain, understand, and use health information as a basis for making correct health decisions and following treatment-related advice (e.g., [1]). Adequate health literacy enables people to become active care recipients and care givers and effectively steer through ever more complex healthcare systems. Health literacy is related to various health-related outcomes, such as the utilization of preventive healthcare services, chronic disease management, and mortality [1,2,3]. Low levels of health literacy are frequently associated with limited risk factor understanding, poor self-management in chronic diseases [4,5,6], and lower adherence to prescribed treatment [7]. Caregivers’ health literacy can also have a profound impact on health outcomes in care recipients, including children [8].

However, it is not well-understood how health literacy affects health outcomes [1]. One pathway may lead from health literacy to health outcomes by means of access to healthcare (for theoretical overview see e.g., [9,10,11,12]). Arguably, patients with lower health literacy may have relatively poorer access to healthcare and therefore achieve relatively worse health outcomes. Although the notion of “access to care” is a complex concept and can be understood within different frameworks (for an overview see e.g., [12]), for the purpose of this study we broadly understand it as the individual’s “ability to position oneself to receive a healthcare service” which can be impeded by high costs of care, inability to schedule an appointment soon enough, lack of transportation, and other factors [13]. The actual utilization of care presumes access and includes the actual realization of healthcare services (e.g., [9]). Access to care, and consequently utilization, is strongly related to health insurance coverage [14]. However, the relationship between health literacy and access to healthcare appears relevant also for the insured patients. A small body of US research that researched the relationship between health literacy and access in insured patients showed that patients with lower health literacy still have relatively more difficulties accessing care [15] and tend to delay or avoid receiving both preventive and curative care [16,17], despite being covered by the insurance. It is hypothesized that, in the US, the rapid expansion and complexity of healthcare services hinders patients’ ability to orient themselves in healthcare systems and hence they may avoid doing so [18]. In the EU, the complexity of health services is also on the rise (e.g., [19]. Unlike in the US, all or nearly all EU residents have comprehensive health insurance, but access to care may still be hindered by co-payments or waiting lists as well as hurdles involving transportation to and from healthcare providers or the increasing healthcare complexity. Arguably, people with lower health literacy may face these obstacles with more difficulties due to their relatively lower socio-demographic capacity to resolve them (e.g., [20,21]), which may in turn lead to delaying or failing to receive care and conversely worse, even detrimental health outcomes. A recent study confirmed that more socially deprived individuals face longer waiting times and are less likely to receive complex emergency care or be admitted for inpatient care but are, on the other hand, more likely to re-attend the emergency department and more likely to die shortly after attendance [22].

The goal of the current study is to explore the association between health literacy and access to healthcare for the first time in Croatia and, to the best of our knowledge, for the first time in the EU context. We use data obtained from face-to-face interviews in a large nationally representative sample of the Croatian population (*n* = 1000) to estimate the level of health literacy and access to care in Croatia. We hypothesize that those with lower health literacy will report relatively more self-perceived barriers to access, also when controlling for other important determinants of access (e.g., [23]).

In addition to exploring the association between health literacy and self-reported access, our paper adds to the literature by reporting, for the first time, the population-wide health literacy levels in Croatia. So far, empirical investigations into health literacy in Croatia were confined to segmented subpopulations [24,25,26,27]. Finally, access to healthcare is rarely analyzed in the Croatian context. This study aims to fill that gap in the literature and examine the socio-demographic disparities in access to care.

## 2. Methods

### 2.1. Data

During April and May 2022, our cross-sectional data were collected by a professional survey agency using a face-to-face interview method (F2F) in a random two-stage stratified nationally representative sample of the general public (age 18+) in all six Croatian regions and five types of settlements by size. A total of 60 interviewers (with the help of 12 supervisors) conducted the interviews in person in respondents’ households. F2F surveys were carried out by computer-assisted personal interviewing; that is, personal interviewing using a tablet device. Technicians were briefed on the content of the questionnaire and were able to provide explanations to respondents. During the survey process, the respondents also had paper cards in front of them with the offered answer scales for convenience and the ease of understanding (e.g., a card with the health literacy measurement tool). Pending their acceptance to participate in the survey, respondents signed informed consent forms. Quality control was carried out during the data collection process (supervisor present during the interview) and after the data collection process (by logical checks of the survey log and return telephone calls to research participants).

### 2.2. Sample

The data from the National Institute of Statistics (population census parameters from 2021) were used to create the structure of the planned sample with a target size of 1000 respondents. The sampling starting points were determined using the relevant data from each of the six regions. The samples included 100 sample points, 10 surveys in each, and the planned/anticipated number of respondents was surveyed in each location. For these 100 sample points, a total of 3604 addresses were visited in order to collect data from 1000 respondents (2604 drop-outs). Within each location/city/region, probabilistic sampling design ensured representativeness of the sample according to gender and age and regional parameters (without islands). The probabilistic nature of sampling was ensured by the use of probabilistic selection procedures and rules, including (1) random selection of sampling starting points, (2) random selection of households, and (3) random selection of a potential respondent in the household.

### 2.3. Variables of Interest

#### 2.3.1. Health Literacy

Health literacy was measured using the Newest Vital Sign (NVS) instrument [28]. NVS is among the most frequently used tools for assessing health literacy [29], and is considered to be valid and reliable for identifying patients with low health literacy [30]. NVS is easy and quick to administer, consisting of an ice cream label and six associated questions, requiring from respondents some simple mathematics and the capacity to interpret basic text. On the basis of correct responses, we categorized patient’s health literacy level such that 0 to 1 correct answer indicates a high likelihood (50% or more) of limited health literacy (labelled limited health literacy); 2 to 3 correct answers indicate a possibility of problematic health literacy (labelled problematic health literacy) while 4 to 6 correct answers indicate adequate health literacy (labelled adequate health literacy) [31].

#### 2.3.2. Access to Healthcare

Access to care was assessed using two instruments. First used was the European Statistics of Income and Living Condition survey item (Eurostat EU-SILC [32]), which estimates the level of self-assessed unmet medical need (e.g., [33]) and measures the share of people who at least once in the previous 12 months felt they needed medical care but did not receive it. This subjective indicator is useful for registering the self-perceived medical needs that do not lead to actual healthcare services utilization and for classifying the self-perceived barriers that prevent individuals with health needs from seeking care [14]. The notion of unmet need translates into access barriers to health care services in four domains: (1) accessibility (could not afford to and too far to travel/no means of transportation); (2) availability (long waiting list); (3) acceptability (could not take time because of work, care for children or for others; fear of doctor/examination/treatment; wanted to wait and see if the problem improves on its own; did not know any good doctor); and (4) other reasons. Respondents could select only one of the offered options. The second question used to measure the self-evaluated ease of accessing healthcare asked respondents to indicate, when they need healthcare services, how easy or difficult is it for them to receive the care they need. Possible responses, using a Likert scale, ranged from 1 (extremely difficult) to 10 (extremely easy).

#### 2.3.3. Explanatory Variables

Health-related factors included having a chronic morbidity (assessed by asking if the respondent suffered from one or more longstanding illness lasting at least six months), calculated body mass index (BMI; calculated using the formula kg/m^2^; conventionally, a BMI of 25.0 or more indicates overweight, a healthy range is from 18.5 to 24.9), self-perceived health (assessed by asking how a person perceives his/her health in general using categories very good/good/fair/bad/very bad), as well as using an EQ5D visual analogue scale (VAS) ranging from 0 to 100 (worst imaginable health—best imaginable health). Demographic and socio-economic (i.e., predisposing) factors included age, gender, and living in an urban area (>25,000 inhabitants). Enabling factors included education level, household income, and living in a single-person household (a proxy for the extent of social support).

### 2.4. Questionnaire Translation

The instruments to measure health literacy (New Vital Sign, NVS) and access (Eurostat’s EU-SILC methodology [32]) were translated to Croatian from English using a validated procedure [34]. First, two translators, fluent in both English and Croatian (Croatian native speakers) and acquainted with the two cultures, made two independent translations from Croatian to English. The first two translators had a basic understanding of the instrument. The two independent translations were fused into a single version by the author. Next, two different translators, again fluent in both English and Croatia (English native speakers) and familiar with both cultures (but unfamiliar with the questionnaire) independently translated back the merged document from Croatian to English. The two back translations were combined into a final version of the instruments by the author and compared to the original English versions of the NVS and EU-SILC question. After confirming that the translations match the original, the questionnaire was finalized.

### 2.5. Analysis

We analyzed the disparities in access and health literacy by stratifying the samples by health-related factors, enabling factors, and predisposing factors. The distributions of the study population were presented as percentages and *p*-values (*p* value of 0.05 was used as a significance threshold) calculated using the chi-square test. All analyses were performed using STATA SE16 (StataCorp LP, College Station, TX, USA). In line with the theoretical model suggested by Lee et al. (2004) [9], health literacy can be considered a determinant of access to care, proxied here by unmet medical need. To test whether health literacy explains a part of the variance in unmet medical need, we performed a multinomial logistic regression with unmet need as the dependent variable, coded as one if respondents answered yes to the questions of whether, in the previous 12 months, they felt as though they needed medical care but did not receive it, zero otherwise. Health literacy level was used as an independent variable (coded by levels—limited, problematic, and adequate) along with control variables. Using a behavioral model of health services use [14,23,35], control variables included predisposing, health need, and enabling factors.

## 3. Results

The sample consisted of 1000 respondents whose socio-demographic and health-related characteristics are presented in Table 1 (mean/share and standard deviations around the mean). The sample was representative of the Croatian population in terms of age and geographical distribution. Most respondents live in towns with more than 25,000 inhabitants and are on average 44.5 years old; females are somewhat more represented, as would be expected. Most respondents attained high school education, which is in line with the relevant national statistics. Reported levels of household income are lower than would be expected when looking at the average monthly net salary of EUR 1000 per employee in Croatia [36], but it is doubtful whether respondents reported their full household income, perhaps due to strategic bias. Health-related data fit well with the relevant national statistics, and 37% of the population suffers from one or more chronic disease, in line with Eurostat data [37]. On average, the population is overweight (judging by the standard cut-off point of 25 for being overweight). The average BMI score is 25.1 and 50.8% of the sampled population has a BMI over 25, which is only somewhat less than national averages published by the Institute of Public Health [38]. Despite the prevalence of chronic conditions, a large majority of the population evaluates their health as good or very good, again in line with EU-wide data [39].

The rates of unmet medical needs obtained in this study are 8.9 per 100 inhabitants (Table 2). The most frequent explanation for unmet medical need and therefore for accessing care is a long waiting list (59.55% of unmet need rates). As expected, the prohibitively high cost of care is not a major issue in Croatia. Surprisingly, however, neither are the distance or the lack of transportation. This is surprising given that rural areas are often under-resourced with healthcare providers (especially the islands, although these were not sampled). On a scale from 1 to 10, the ease of receiving care when needed is evaluated with an average score of 7.33.

There is a statistically significant difference in the unmet medical need in subpopulations stratified by health and age (Table 2). Those that have a chronic condition are 75% more likely to have an unmet medical need. The odds of having an unmet medical need are 87% higher in the oldest age group than in the youngest. Seemingly, those in the greatest need face (or receive facing) relatively larger obstacles when accessing care. Encouragingly, however, unmet medical need does not significantly vary by household income or the degree of urbanization; education, gender, and living in a single-person household also do not make a difference for the level of perceived unmet medical need.

The majority of the Croatian population has a limited or problematic level of health literacy (53.1%; Table 3). The average NVS score of 4,05 out of 6 somewhat glooms the fact that 36.9% of the population correctly answered only 1 to 3 questions, signaling that, for a part of the population, health literacy is at very low levels and could be a major obstacle to maintaining and improving their health. Respondents with limited or problematic health literacy are more likely to perceive their health as bad or very bad, evaluate their health as lower than 60 on a VAS scale and report having one or more chronic diseases than those with adequate health literacy (*p* < 0.05 and *p* < 0.1; Table 3). The odds of having one or more chronic conditions are about 20% higher for people with limited health literacy than for respondents with adequate health literacy. The odds of perceiving health as bad or very bad relative to fair/good/very good health are more than double for respondents with limited health literacy than in respondents with adequate health literacy. These results are in line with US research (e.g., [40]). Furthermore, the odds of adequate health literacy are more than double in the youngest respondents relative to the oldest. Although health literacy levels are not related to respondents’ gender (*p* > 0.1), lower rates of health literacy are found in persons living with lower income and education living in larger urban areas (*p* < 0.05). The odds of adequate health literacy are 30% lower in those with elementary education relative to more highly educated. The odds of limited health literacy are about 14% higher in the poorest households relative to the richer households.

In summary, Table 2 and Table 3 already reveal a statistically significant association between a self-received level of unmet medical need and persons’ health literacy. Table 3 shows that persons reporting unmet medical need in the previous year are 53% more likely to have limited health literacy than adequate health literacy (*p* = 0.018). Using a simple logit regression with binomial variable unmet need (where 1 = yes, 0 = no) as the dependent variable, we show that this finding holds when controlling for the enabling, predisposing, and health factors as well (Table 4). Controlling for other important determinants of access, NVS is significantly associated with unmet need, a proxy for access to care. In other words, when controlling for health factors (chronic disease prevalence and BMI), predisposing factors (age, gender, and living in an urban area) and enabling factors (household income and living in a single-person household), the NVS score is in a negative and statistically significant relationship with unmet need—the higher the NVS score, the lower the probability of reporting unmet medical need. This relationship holds when we insert interaction terms (e.g., age × chronic disease) as well as when the NVS score is transformed into a categorical variable with health literacy levels (adequate, limited, and problematic).

## 4. Discussion

Our main results reveal that the health literacy is limited or problematic in the majority of the Croatian population. At the same time, the rate of unmet medical need—a proxy for access to care—is 8.9 per 100 inhabitants. Those rates are higher in the group with limited and problematic health literacy, also confirmed by the logit regression. Controlling for predicting factors, lower health literacy is significantly related to a higher level of unmet medical need.

With respect to health literacy levels in Croatia, our findings are similar to previous findings [41], which used also used the NVS instrument to measure health literacy, albeit in a small convenience sample of 100 hospital patients in Croatia, and found the 58% of the sample had limited or problematic health literacy. The small sample notwithstanding, it is not surprising that already hospitalized patients who are on average older than the general public perform somewhat worse than the general public on a health literacy test. Internationally, our results are also supported by previous NVS-based health literacy research, which suggests relatively high problematic or limited health literacy in different jurisdictions and subgroups of the populations, such as the elderly (e.g., [42]), caregivers [43], and in cardiovascular disease patients [44].

With respect to the levels of unmet need, it has to be recognized that although health insurance coverage is provided to all residents in Croatia (and the EU), a certain percentage of the population still reports unmet medical needs. In spite of insurance coverage, for one reason or the other, not all who seek care can access the healthcare provider. In Croatia, waiting lists are the most often cited reason thereof, while geographical location and costs are not as problematic. This signals that the solution to reducing unmet medical need lies mainly with the healthcare system organization and management as well as with providers themselves (for more debate on waiting lists see e.g., [45,46]). The same was previously argued regarding bridging the health literacy gap. The negative association between health literacy and unmet need (lower self-evaluated access to care) is statistically significant. This relationship between unmet need and health literacy is likely a single-direction relationship, with health literacy levels affecting the unmet medical need (not the other way around, both from the theoretical [9] as well as purely logical perspective). Although we cannot claim a causal relationship between the two variables (based on our results), our findings support the future research of quantifying the association between health literacy and health outcomes by means of a mediating factor: access to healthcare. This is especially relevant in Croatia and other Central European jurisdictions where health outcomes generally lag behind the Western EU-member states. For instance, mortality rates in prevalent illnesses such as cancer and cardiovascular diseases in Croatia are highest or among the highest in the EU [37,47]. Previous research showed that lower health literacy is associated with detrimental health outcomes (e.g., [1,3]), and given that 53% of the Croatian population has limited or problematic health literacy and that the rates of unmet medical need are highest in these population subgroups, health literacy needs to be considered in this wider context.

These have important policy-making implications. Foremost, it has to be recognized that limited and problematic health literacy is commonly reported in the literature, and as such, it seems wise to consider it as a fact when designing and delivering health interventions (e.g., self-management education in chronically ill) as well as when developing and advocating new healthcare paradigms that promote equity of care and improve quality if we want our interventions to work as intended. Patient-centered care, the innovative approach to modernizing and transforming the organization of healthcare services in order to adjust to the modern-day health problems (such as the growing prevalence of chronic conditions or the growing incidence of cancer) [48] presupposes, among else, active involvement of patients and their families in modernizing the design and the delivery of our healthcare services. Patients and their families are expected to actively participate in increasingly individualized treatment decision-making [49]. We need to be asking whether patient-centred care can deliver what it promises if more than half our population cannot interpret relatively simple instructions posted on an ice-cream container. In clinical settings, NVS can precisely be used by providers to detect when their communication should be adjusted to the patient’s (low) health literacy level. Unlike age or other socio-demographics, the healthcare system can (and should) affect both the levels of health literacy as well as adjust its approach to individuals with lower health literacy. It was argued before e.g., [16] that for patients with lower numeracy skills, for instance, providers should explain available treatment plans without using complex terminology and numerical jargon. This implies shifting the focus from health literature individuals to health literacy of healthcare organizations. Already decades ago, calls were made to shift the focus of healthcare systems toward rethinking and developing modes of care delivery that do not require advanced health literacy skills at all [50] with the goal of increasing safety, timeliness, efficacy, and effectiveness of health services [51]. Such universal precautions imply designing healthcare services to fit any patient, without the need of knowing which patients actually have low or high health literacy may be the best way to ensure that patients have all the information needed for making appropriate health-related decisions [52]. All this requires conscious effort. In Croatia, health literacy is finally recognized in the official state documents, although for now only declaratively, although the health literacy of organizations is not mentioned as of yet. The new Croatian Healthcare development plan [53] highlights health literacy as a mean to empowering patients and citizens as a means of attaining better health outcomes. It remains to be seen whether and how health literacy will be operatively addressed in the design of new interventions. Unmet medical need reported in this study is considerably higher than the rate for Croatia obtained from Eurostat’s last available 2019 EU-SILC data (4.2 per 100; [32]). Long waiting lists were the most frequently reported reason for unmet need at the EU-28 level, according to Eurostat, the same as the findings we report here. The difference in the rates of unmet need between Eurostat’s and our data could be attributed to the COVID-19 pandemic, which in 2021 and the beginning of 2022 gripped Croatia, as well as the rest of Europe. Although at the time of face-to-face data collection there was no lock down in Croatia, in the 12 previous months, there were periods when the healthcare system was less available to patients, either due to lockdowns or the number of COVID-related patients overburdening the system. This potentially contributed to the level of unmet need, especially in the domain of waiting lists, and explain why Eurostat’s data on the level of unmet medical need available for 2019 [32] reported levels of unmet need twice lower than what we find in 2022. If this is indeed the case, then it can be argued that the COVID-19 pandemic doubled the unmet medical need in Croatia. However, the EU-SILC data are not available from Eurostat for 2020–2022, so we cannot confirm or refute this hypothesis.

Lastly, long waiting lists, as already argued, are in the domain of the healthcare system, and while a certain level of waiting list is a useful tool for mediating demand-side moral hazard, lists that are too long prohibit access to care. This is especially true for those patient groups with highest healthcare demand: the elderly and the frail. Reforms that will address waiting times will help address unmet medical needs, but policy-makers should be particularly vigilant in designing additional policies aimed particularly at those with relatively poor health and old age [54].

One of the main limitations of the study is its cross-sectional design, which did not allow for causal relationships between the main variables of interest to be explored. We also could not explore the association between low health literacy and (other) important health-related outcomes in more detail since these were not available in our data. Future research, with more detailed longitudinal data (available from large population-based panels), could explore the relationship between health literacy and health outcomes accounting for the mediating effect of unmet medical need. Furthermore, although the NVS instrument used to measure health literacy demonstrated its acceptability to patients and validity in different settings, as well as correlations to more elaborate health literacy screening tools (e.g., [30]), it primarily measures numeracy and reading skills [28,55], suggesting that NVS is not necessarily an overall measure of health literacy. Future research may explore the relationship between access to care and health literacy measured using another validated tool. Finally, the COVID-19 pandemic could have affected the results regarding the unmet medical need. It can be recommended to track the level of unmet need in the future and use these results to check the impact of the COVID-19 pandemic on the level on unmet medical need.

## 5. Conclusions

Our study showed that limited health literacy is prevalent in Croatia and is associated with higher rates of unmet medical need, i.e., poorer access to care. Lower health literacy is found in groups of older individuals, individuals who are in relatively poorer health, living with lower income, and with poorer educational attainment. Unmet medical need, fuelled primarily by long waiting lists, is relatively higher in older individuals and individuals with poorer health status. It would be advisable to simplify health services and improve health education with a potential of improving the access to care and (consequently?) health outcomes, as well as to design policies which would reduce waiting times in Croatia since these contribute most to the levels of unmet medical need.

## Figures and Tables

**Table 1:** Socio-demographics and health-related data.

Variable	Variable Category	Mean/Share	St. Dev.
**Age**	Ranging from 18 to 89	44.48	16.9
	<30	23.8%	
	30–50	36.2%	
	50–70	31.9%	
	>70	8.1%	
**Education**	Elementary	7.6%	
	High school	67.7%	
	University or higher	24.7%	
**Gender**	Female	53.0%	
**Town size**	>25,000 inhabitants (urban area)	60.8%	
**Household income**	<EUR 733	52.0%	
	EUR 734–EUR 1200	27.6%	
	>EUR 1200	20.3%	
**Having one or more chronic diseases**	Yes	37.0%	
**BMI (kg/m^2^)**	Mean (range)	25.10 (min 15.95–max 66.4)	4.52
	>25	50.8%	
**Self-rated health**	Ranging from 1 very good to 5 very bad	1.88	
	Good and very good	43.5%	
	Fair	49.8%	
	Bad or very bad	6.8%	
**Visual analog scale (VAS)**	mean	81.40	20.90
**Wellbeing**	ranging from worst to best imaginable	7.48	2.03
**Household composition**	Single-person household	15.4%	
	Multi-person household	84.6%	

**Table 2:** Unmet medical need.

Variables	Variable Category	Share	St. Dev.	
Unmet medical need * (% yes)		8.90%	0.28	
**Accessibility**	Could not afford to.	7.84%		
	Too far to travel/no means of transportation.	2.25%		
**Availability**	Long waiting list.	59.55%		
**Acceptability**	Could not take time because of work. Care for children or for others.	6.74%		
	Fear of doctor/examination/treatment.	0%		
	Wanted to wait and see if the problem improves on its own.	16.85%		
	Did not know any good doctor.	1.12%		
	Other reasons.	5.65%		
***When you need healthcare services. how easy or difficult is it for you to receive the care you need?***		7.33	2.16	
	1	1.7%		
	2	1%		
	3	3.3%		
	4	4.9%		
	5	9%		
	6	11%		
	7	15.8%		
	8	20.6%		
	9	14.1		
	10	18.6		
**Unmet medical need stratified by health factors, enabling factors and predisposing factors**		**No unmet medical need**	**Unmet medical need**	
**Self-rated health**	Good and very good	94%	6%	*p* < 0.001
	Fair	89%	11%	
	Bad or very bad	65%	35%	
**Having one or more chronic diseases**	No	95%	5%	*p* < 0.001
	Yes	84%	16%	
**BMI (kg/m^2^)**	Equal or less than 25	92%	8%	*p* > 0.13
	>25	90%	10%	
Visual analog scale (VAS) **	<60	76%	24%	*p* < 0.001
	>59 and <80	86%	14%	
	>79	96%	4%	
**Gender**	Female	91%	9%	*p* = 0.85
	Male	91%	9%	
**Household income**	<EUR 733	90%	10%	*p* = 0.47
	EUR 734–EUR 1200	92%	8%	
	>EUR 1200	92%	8%	
**Place of residence**	<25,000 inhabitants	91%	9%	*p* = 0.87
	>24,999 inhabitants	91%	9%	
**Age**	<30	96%	4%	*p* < 0.001
	30–50	94%	6%	
	50–70	88%	12%	
	>70	77%	23%	
**Education**	Elementary	87%	13%	*p* = 0.38
	High school	92%	8%	
	University or higher	91%	9%	
**Household composition**	Single-person household	89%	11%	*p* = 0.36
	Multi-person household	91%	9%	
**Health literacy—NVS score**	Limited health literacy	87%	13%	
	Problematic health literacy	91%	9%	
	Adequate health literacy	93%	7%	

Note: * Exact questions: “Was there any time during the last 12 months when. in your opinion. you needed a medical examination or treatment for a health problem but you did not receive it? (% yes)”. ** VAS cut-off ranges depict the distribution of the scores on the VAS scale (0–100), with scores lower than 60 noted in 18.3% of the respondents, between 60 and 80 in about 25% of respondents, and the rest higher than 80 score (81.4 being the average score).

**Table 3:** Health literacy.

Variables	Variable Category	Share	St. Dev.		
**NVS score**	Average number of correct answers ranging from 1 to 6.	4.05	1.57		
**NVS score (sum of correct answers)**	NVS score ∑1	7.2%	0.33		
	NVS score ∑2	13.0%	0.37		
	NVS score ∑3	16.7%	0.37		
	NVS score ∑4	16.4%	0.43		
	NVS score ∑5	24.5%	0.41		
	NVS score ∑6	22.2%	0.44		
**Health literacy—NVS score**	Limited health literacy	20.1%	0.45		
	Problematic health literacy	33.0%	0.47		
	Adequate health literacy	46.9%	0.49		
**Health literacy stratified by health factors. enabling factors and predisposing factors**		**Limited** **health literacy**	**Problematic** **health literacy**	**Adequate** **health literacy**	
**Self-rated health**	Good and very good	19%	34%	47%	*p* = 0.045
	Fair	20%	35%	50%	
	Bad or very bad	34%	26%	40%	
**Having one or more chronic diseases**	No	18%	35%	47%	*p* = 0.094
	Yes	23%	35%	47%	
**BMI**	Equal or less than 25	42%	48%	53%	*p* = 0.024
	>25	58%	52%	47%	
**Visual analog scale**	<60	33%	28%	38%	*p* < 0.001
	>59 and <80	20%	33%	47%	
	>79	17%	34%	49%	
**Gender**	Female	22%	34%	44%	*p* = 0.3
	Male	19%	35%	49%	
**Household income**	<EUR 733	23%	33%	44%	*p* = 0.03
	EUR 734–EUR 1200	19%	35%	52%	
	>EUR 1200	15%	38%	48%	
**Place of residence**	<25,000 inhabitants	17%	32%	51%	*p* < 0.001
	>24,999 inhabitants	25%	35%	41%	
**Age**	<30	15%	33%	51%	*p* = 0.01
	30–50	21%	30%	49%	
	50–70	20%	35%	45%	
	>70	31%	38%	31%	
**Education**	Elementary	32%	26%	42%	*p* = 0.01
	High school	21%	33%	46%	
	University or higher	14%	36%	50%	
**Unmet medical need**	No	19%	33%	48%	*p* = 0.018
	Yes	30%	35%	35%	

**Table 4:** Multinomial logistic regression.

Dependent Variable: Unmet Medical Need	Coefficient	Std. Err.	*p* > |z|	95% Confidence Interval	
**NVS score ^1^**	−0.17	0.07	0.02	−0.32	−0.03
**Chronic disease (1 = yes)**	1.00	0.28	0.00	0.45	1.54
**Age**	0.03	0.01	0.00	0.01	0.05
**Education ^2^**	0.29	0.23	0.21	−0.17	0.74
**Place of residence ^3^**	0.01	0.24	0.98	−0.47	0.48
**Gender (1 = female)**	0.05	0.24	0.83	−0.42	0.52
**Household income ^4^**	0.08	0.16	0.62	−0.24	0.40
**BMI (1 = >25)**	−0.12	0.25	0.63	−0.60	0.37
**Single-person household (1 = yes)**	−0.13	0.31	0.67	−0.75	0.48
**Constant**	−4.37	0.89	0.00	−6.11	−2.62
**Pseudo R^2^ = 0.09**					

Note: ^1^ range 1–6, where 6 is the highest attainable score; ^2^ specified as a categorical variable where 1 = elementary, 2 = middle school, 3 = university level of education; ^3^ specified as a dummy variable where 1 = 24,999 inhabitants or more, 0 = less than 24,999 inhabitants; and ^4^ specified as a categorical variable where 1 = <EUR 733, 2 = EUR 734–EUR 1200, 3 > EUR 1200.

## Data Availability

The data presented in this study are available on request from the corresponding author. The data are not publicly available due to requirements of the funding body.
